# Lifestyle Matters for maintenance of health and wellbeing in people aged 65 years and over: study protocol for a randomised controlled trial

**DOI:** 10.1186/1745-6215-14-302

**Published:** 2013-09-21

**Authors:** Kirsty Sprange, Gail A Mountain, John Brazier, Sarah P Cook, Claire Craig, Daniel Hind, Stephen J Walters, Gill Windle, Robert Woods, Anju D Keetharuth, Tim Chater, Kath Horner

**Affiliations:** 1 School of Health and Related Research (ScHARR), University of Sheffield, Regent Court, Regent Street, Sheffield S1 4DA, England, UK; 2 Centre for Health and Social Care Research, Montgomery House, 32 Collegiate Crescent, Collegiate Campus, Sheffield Hallam University, Sheffield S10 2BP, England, UK; 3 Dementia Services Development Centre, Institute of Medical and Social Care Research, Prifysgol Bangor University, 45 College Road, Bangor, Gwynedd, Wales, UK; 4 NHS Sheffield, 722 Prince of Wales Road, Sheffield S9 4EU, England, UK

**Keywords:** Lifestyle Matters, Psychosocial Intervention, Prevention, Older adults, Quality of life, Wellbeing, Mental health, Mental wellbeing

## Abstract

**Background:**

Healthy, active ageing is strongly associated with good mental wellbeing which in turn helps to prevent mental illness. However, more investment has been made into research into interventions to prevent mental illness than into those designed to improve mental wellbeing. This applied research programme will provide high quality evidence for an intervention designed to improve and sustain mental wellbeing in older adults.

**Methods/Design:**

This study was a multi-centre, pragmatic, two-arm, parallel group, individually randomised controlled trial to determine the population benefit of an occupational therapy based intervention for community living people aged 65 years or older. Participants (n = 268) will be identified in one city in the North of England and in North Wales through GP mail-outs, signposting by local authority, primary care staff and voluntary sector organisations and through community engagement. Participants will be randomised to one of two treatment arms: an intervention (Lifestyle Matters programme); or control (routine access to health and social care). All participants will be assessed at baseline, 6 and 24 months post-randomisation. The primary outcome, which is a person reported outcome, is the SF-36 Mental Health dimension at six months post randomisation. Secondary outcome measures have been selected to measure psychosocial, physical and mental health outcomes. They include other dimensions of the SF36, EQ-5D-3L, Brief Resilience Scale, General Perceived Self Efficacy Scale, PHQ-9, de Jong Gierveld Loneliness Scale, Health and Social Care Resource Use and the wellbeing question of the Integrated Household Survey 2011. A cost effectiveness analysis will investigate the incremental cost per Quality Adjusted Life Years (QALYs) of the Lifestyle Matters intervention compared with treatment as usual.

**Discussion:**

The questions being posed through this research are important given the increasing numbers of older people, pressure on the public purse and the associated need to support good health in the extended lifespan. The proposed trial will determine the clinical and cost effectiveness of the intervention delivered in a UK context. The results will support commissioners and providers with decisions about implementation.

**Trial registration:**

Current Controlled Trials ISRCTN67209155

## Background

Mental wellbeing in later life is strongly associated with healthy, active ageing, which, in turn, helps to prevent mental illness [1-3]. It has been established that mental wellbeing can be promoted by participation in meaningful activities/occupations and by active engagement with life [4-6]. Life changing events in later years, such as diagnosis of a long term health problem or bereavement, can lead to reduction in engagement with life which can result in eventual decline in mental wellbeing. Prevention of this decline could lead to reduced need for health and social care services and promote the re-engagement of people with their local communities. Far more investment, however, has been made into research into interventions to prevent mental illness than into those designed to improve mental wellbeing [7]. This programme will provide high quality evidence for an intervention designed to improve and sustain wellbeing, thereby contributing towards redressing the imbalance.

The Health and Social Care Act 2012 introduced radical changes to improve care provision in England, including the abolition of Primary Care Trusts and the introduction of clinical commissioning groups and an NHS Commissioning Board referred to as NHS England [8]. Further legislative change through the proposed Care Bill (2013) in England, currently awaiting parliamentary approval, and the draft Social Services and Well-Being (Wales) Bill will also help drive co-ordination of health and social care services, promote health and wellbeing and support independent community living [9,10]. This new integrated approach to health and social care service provision over the lifespan could be harnessed to garner greater investment in community based social interventions to prevent decline in health and mental wellbeing and isolation in older people.

A systematic review of evidence to support UK National Institute for Health and Care Excellence (NICE) guidance on interventions to promote good health and wellbeing in older people confirmed that a US health promoting intervention (Lifestyle Redesign® The American Occupational Therapy Association, AOTA Press, USA) provided robust effectiveness and cost effectiveness evidence [11,12]. The intervention was able to significantly enhance the physical and mental health, occupational functioning and life satisfaction of community-living older adults [13,14]. Approximately 90% of the post intervention therapeutic gain was retained at follow-up six months later [15]. Furthermore, Clarke *et al.* (2011) found the base case cost per quality adjusted life year (QALY) was within the range considered cost effective by NICE [16]. A feasibility study conducted in Sheffield with older adults aged 60 to 92 years tailored the intervention to a UK context and determined that delivery was possible [17]. The result of this initial work was the Lifestyle Matters intervention. The success of the intervention (which is a mix of facilitated group and individual sessions) is based on positioning the older person as the expert, thereby facilitating improved confidence, and associated positive behaviours. The intervention focuses on enabling participants to undertake new or neglected activities in the community, make lifestyle choices, undertake personal goal setting and be active in their own personal development. The overall goal is to promote long term change and associated psychological benefit. The feasibility study was found to harness the resources of older people and use of community facilities rather than fostering reliance upon statutory services [18].

Lifestyle Matters is currently recommended for implementation within UK NICE guidance (2008) and can be located on the NHS evidence site for Quality, Productivity and Prevention (QIPP) where it is stated that ‘results of replication are not yet determined’ [19]. The systematic review which underpinned the NICE Guidance rated the pilot study as being ‘sound qualitatively’ but we remain reliant upon the results of a US randomised controlled trial to provide population-based evidence for an intervention that is highly dependent upon cultural context [12]. Despite support with implementation and the extensive need that exists amongst older people, the UK response to the NICE Guidance has been inconsistent. The Lifestyle Matters intervention can be delivered by either health or social care and by the statutory or third sector and, therefore, ‘falls’ between different providers, tending not to be prioritised. Also the only evidence to support implementation of a UK based programme (for example, the skills and competencies of service providers and UK costs for commissioners of services) is limited to that identified through the feasibility study.

The proposed research provides the opportunity to determine the clinical and cost effectiveness of the Lifestyle Matters intervention in a UK context, thereby also adding to the national and international evidence base. We aim to establish how mental wellbeing, self-efficacy and resilience can be supported in community living people aged 65 years and older. We will examine the underlying mechanisms that can promote healthy ageing and determine the long term sustainability of the intervention. Incremental cost effectiveness will be explored using cost effectiveness analysis and cost utility analysis in terms of cost per QALY of the Lifestyle Matters intervention compared to usual care. The results will support commissioners and providers with information to underpin decisions about implementation.

## Methods/Design

### Trial design

**Figure 1:**
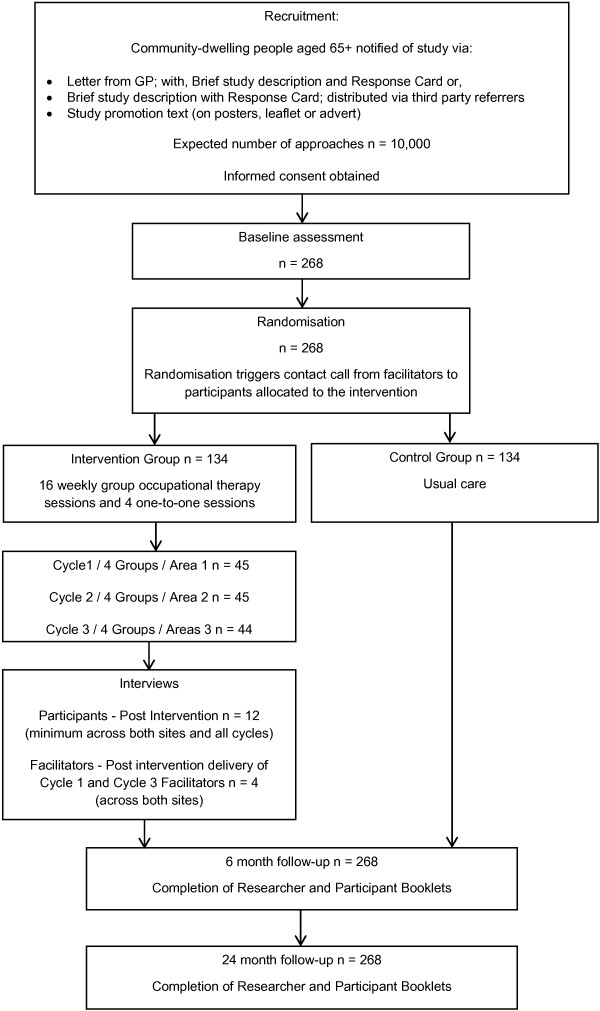
Lifestyle Matters CONSORT diagram.

The study is a multi-centre pragmatic, two-arm, parallel group, individually randomised controlled trial, to investigate the implementation of a psychosocial intervention, Lifestyle Matters, which aims to promote mental wellbeing in people aged 65 years or older. Participants recruited to the study will be randomly allocated to receive either the Lifestyle Matters programme intervention in addition to usual care or to usual care only which is defined as routine access to health and social care resources. All participants will complete the same battery of outcome measures at baseline, 6 months and 24 months to ascertain the benefits that might be derived from participation immediately after cessation of involvement and over time. The trial will be delivered at two study sites, one city in the North of England and in North Wales. The trial will adhere to the Medical Research Council (MRC) framework for the evaluation of complex interventions [20]. A CONSORT-style flow diagram is provided in Figure 1[21]. The study has been approved by the South Yorkshire Research Ethics Committee, the National Institute for Social Care and Health Research (NISCHR) Permissions Co-ordinating Unit in Wales, Sheffield Health and Social Care NHS Foundation Trust, NHS Sheffield, Sheffield Teaching Hospitals NHS Foundation Trust, Betsi Cadwalador University Health Board (BCUHB) and Sheffield City Council. The trial is registered with Current Controlled Trials, reference number ISRCTN67209155.

#### Aims and objectives

The primary aim of the study is to identify how mental wellbeing, self-efficacy and resilience can be supported in people aged 65 years or older by:

1. Evaluating the clinical and cost effectiveness of a psycho-social intervention to promote healthy ageing (Lifestyle Matters) compared to usual care.

2. Examining the nature of the underlying mechanisms that might promote self-efficacy and resilience.

3. Determining the long term sustainability of the intervention and any associated treatment effects.

#### Intervention

Groups of 8 to 16 participants will attend 16 weekly facilitated sessions over four successive months at a local community venue. During these sessions participants will undertake activities as agreed between group members, which may take place at the weekly venue or within the wider community setting. Didactic sessions relevant to the needs of specific members will also be woven into the programme to enhance participants’ knowledge of how to overcome barriers to active engagement. Each participant will be offered four individual sessions (one approximately every four weeks) with one of the facilitators for the purposes of pursuing personal goals. The content of the intervention includes (but is not limited to) the following themes which are fully documented in the published manual [22].

a) Beginnings - a celebration of achievements (mandatory)

b) Activity and Health (mandatory)

c) Growing older - changing patterns of activity goal setting (mandatory)

d) Maintaining and improving mental wellbeing

e) Maintaining physical wellbeing

f) Occupation in the home and community

g) Safety in and around the home

h) Personal circumstances

i) Endings

Mountain *et al.* (2008) found that the Lifestyle Matters programme could be delivered by non-occupational therapy trained staff. Intervention facilitation will, therefore, be conducted by equivalent NHS Agenda for Change (AfC) Band 4 staff, for example, health trainers, health champions or occupational therapy support workers who are specifically recruited to the study. Two facilitators will deliver each group with weekly supervision of their work being provided by trained occupational therapists. All facilitators and supervisors will receive the same two day training programme delivered by the original author of the Lifestyle Matters manual and receive an accompanying CD-Rom. This will support intervention fidelity by enabling those delivering the complex intervention to learn about the programme and work together prior to commencement. The training will have components of group work, didactic teaching, reflective exercises, role playing and use of scenarios.

#### Control arm

Participants randomised to the control arm will be asked to continue with usual care defined as accessing health and social care acute and community services as appropriate to meet their needs. Those allocated to the control arm will receive a Lifestyle Matters information leaflet at the end of the study period (24 months post randomisation), to try and prevent ‘resentful demoralisation’ as a consequence of non-involvement. The information leaflet will be derived from the published manual and will include signposting to local groups and services.

#### Welsh speakers

Groups will be offered in English, Welsh or bi-lingual, where agreed by the group. The recruitment of Welsh speakers will investigate population differences, acceptability and adherence to the intervention across nationalities. Essential study documentation will, therefore, be translated into Welsh by Bangor University. To ensure standardisation across sites, all documents, including any validated and non-validated questionnaires, not already available in Welsh will also be forward-back translated (English-Welsh-English) with the back translation being compared to the original English for harmonisation and to clarify anomalies. A number of the patient reported outcome measures are already available in Welsh, including the SF-36, EQ-5D-3 L, BRS and de Jong Gierveld Loneliness scale.

### Participant recruitment

A number of geographical areas will be identified in a city in the North of England and in North Wales for recruitment and subsequent intervention delivery. Recruitment and intervention delivery will be in three cycles, each cycle being cited in a community location where the recruited participants reside. The intention is to recruit enough participants to hold two groups of 6 to 18 participants in each location. Older adults aged 65 years and over who are living in their own home or with others will be identified through targeted General Practitioner (GP) mail-outs; signposting by health and social care staff who are likely to come into contact with older people in their work; community based voluntary sector organisations for older people and through community engagement and advertisements. A range of marketing materials will be used to advertise the study and will be made available in English and Welsh.

The main risk to recruitment is the GP mail-outs where there is little control over who responds, leading to two issues. Firstly, those who have self-identified a need or are already confident enough to attend an intervention like Lifestyle Matters and, secondly, attracting only individuals who are already active in their personal life and within their community. To reduce these risks, advice will be sought from local authorities and primary care on potential areas at both sites which currently have limited or less access to community services and other research-based opportunities. It is also anticipated that regardless of individual circumstances, those who register an interest in the study will have a personal reason for doing so.

It is anticipated that the number and availability of GPs will differ in each geographical location and may introduce risk to recruitment. This will pose a greater challenge in North Wales, which predominantly consists of smaller, rural community GPs. In the North of England site, which is a large city, there is anticipated to be enough GP surgeries to conduct recruitment for two groups in each cycle. Assistance with recruitment will, therefore, be sought from the Primary Care Research Network (PCRN) in England and the National NISCHR in North Wales both of which regularly work with research active GPs.

Part of the Lifestyle Matters programme is to encourage and support participants in arranging their own transportation to and from the weekly meetings; therefore, challenges may arise regarding accessibility of venues. This is particularly relevant for participants living in rural areas with limited access to public transportation. Community venues will therefore be selected for their centrality in a geographical area and accessibility by local public transport, including buses and trams where possible, and have adequate parking facilities. In North Wales it is anticipated that although some participants will be expected to live in rural or remote areas, these individuals will already be managing their transport needs; for example, many of these individuals will be car owners. However, requests by participants for support with transportation will be managed on a case-by-case basis.

#### Participant eligibility

All participants will need to display reasonable cognitive function to be able to participate in this group-based intervention as evidenced by a score of 0 to 7 on the Six Item Cognitive Impairment Test (6CIT) [23]. The 6CIT is a simple test of cognition, which shows greater sensitivity for milder dementia than, for example, the Mini Mental State Examination (MMSE) [24]. They will also need to be living independently or in sheltered accommodation, alone or with others and be able to converse in English or Welsh.

#### Participant screening

Participants will register an interest in the study by returning, to the Research Team, a pre-paid response card which is enclosed in the GP mail-out or given to the participant by the direct referrer. Participant screening will then be undertaken in two stages. Stage 1 is First Contact Screening, which will be conducted when a response card is received by the research team who then telephone the participant and ask them to confirm their age, accommodation status and establish whether they are able to converse in English or Welsh. If they are eligible to proceed, an eligibility assessment/baseline interview is arranged which is Stage 2 of the screening process.

The participant will be sent a copy of the participant information sheet approximately one week prior to the Eligibility Interview. During this face-to-face assessment they will be asked to complete the 6CIT [23]. If the individual is eligible, they will have three options. Option one involves the assessor working through the participant information sheet, taking consent and delivering the baseline assessment consisting of a battery of questionnaires. Informed consent will be obtained from each participant. The participant is then randomised to the study and notified of their study arm allocation by a member of the research team (not the assessor). Option 2 will allow the participant further time to consider their decision with the researcher agreeing to future contact with the participant. Option 3 allows the participant to withdraw their interest in taking part in the study.

#### Participant safety

If a potential participant is found not eligible during Stage 2 of screening, based on a significant score of 8 or more on the 6CIT, the interview will be suspended. A significant score is an indicator of mild cognitive impairment. The assessor will inform a designated member of the research team (a registered heath professional), who will telephone the participant to discuss the 6CIT score, its implications and to signpost the individual to appropriate services. If telephone contact is not possible within one week of the Eligibility Interview, the participant will be sent a letter including the details outlined for the above telephone contact. Individuals found to have a significant score on the PHQ-9 depression scale or those whose general behaviour raises concerns during baseline assessment, or 6 and 24 month follow-up, will also be referred to the designated health professional for review and potential further contact.

As part of the recruitment process, participants will be asked to state any current medical conditions which may affect their ability to take part in activities undertaken as part of the intervention. Although the facilitators will conduct on-going monitoring of participants and their involvement in group activities, the participants are ultimately responsible to make independent decisions about their level of involvement in activities. Locations for intervention delivery will be assessed for health and safety, including appropriate access, and warm, appropriate facilities including kitchen and accessible toilets.

#### Withdrawal

Participants will be free to withdraw at any time without giving a reason. However, where possible the reason for discontinuation will be recorded. If a participant withdraws during the study period, data already collected prior to withdrawal will be retained and used for the purposes of the study.

### Outcome measures

Participants in both the intervention and control arms will be asked to complete the same series of patient reported outcome measures at the same time points. The measures will be presented in the form of two booklets at baseline, 6 month and 24 month follow-up. The first is the Assessor Booklet, which the researcher will complete on behalf of the participant and will consist of the following questionnaires:

• SF-36 is a widely used validated reliable measure of quality of life, functional health and wellbeing [25].

• Health and Social Care Resource use questionnaire.

The second is the Participant Booklet which will be self-completed where possible and will consist of the following questionnaires:

• Wellbeing Question from the Integrated Household Survey 2011 is a life satisfaction measure developed by the Office of National Statistics (ONS) [26].

• EQ-5D-3L is a widely used validated measure of health outcomes preferred by NICE in its reference case, with UK specific preference weights [27-29].

• Brief Resilience Scale is a reliable measure for assessing resilience and ability to bounce back or recover from stress [30].

• De Jong Gierveld Loneliness Scale is a validated instrument and reliable for measurement of overall, emotional and social loneliness [31].

• General perceived Efficacy Scale (GSE) assesses self-beliefs used to cope with a variety of demands in life, that is, the belief that one’s actions are responsible for successful outcomes [32].

• PHQ-9 is a validated measure of mood and anxiety, and is widely used in primary care in the UK [33].

Participants will also be asked to complete a socio-demographics questionnaire at baseline.

All six month follow-ups will be completed face-to-face with participants by a blinded assessor. It is anticipated that due to the nature of the intervention that participants, although asked not to reveal the study arm allocation, may inadvertently unblind assessors. Any knowledge of study arm allocation for each participant will, therefore, be recorded at 6 and 24 month follow-up by the blinded assessor. At 24 month follow-up the blinded assessor will deliver and complete the Assessor Booklet by telephone. The Participant Booklet will be sent by post to the participant for self-completion, including a return pre-paid envelope. Assistance will be provided to participants where a need is identified, for example, large print versions of questionnaires, telephone assistance or a home visit.

Due to the length of time between baseline and final assessment, a significant risk will be the potential for deterioration in general health and, in particular, cognitive capacity for some participants. If the accommodation needs of a participant have changed during this time, for example, a need to enter a residential home, this will not prevent follow-up; however, a move to a nursing home will be managed on a case by case basis. Although there is no intention to conduct the 6 Item Cognitive Impairment Test with participants before the 24 month follow-up, the 6CIT may be repeated to record any changes in cognitive function.

### Randomisation

The Sheffield Clinical Trials Research Unit (CTRU) and Bangor University will oversee randomisation. To ensure that assessors are blinded to group allocation, other designated members of the research team will complete randomisation. This will be via a secure remote web-based system which will allocate each participant a unique identification number. Details entered into the system will include confirmation of signed consent. Participants will then be randomly allocated to either the intervention (n = 134) or usual care (n = 134) arm of the trial. In the event of a couple both consenting to take part, the pair will be randomised as a couple and not separately, that is, to either get the intervention, or to both get usual care.

The randomisation schedule will be computer generated, stratified by site and random permuted blocks of variable size will be used to ensure enough participants are allocated evenly to each arm of the trial at each site. The participant’s GP will be notified of their involvement in the study. Participants randomised to the control arm of the trial will be informed by telephone and sent a letter confirming their allocation to keep as a record. Participants allocated to the intervention arm of the trial will first be sent a letter confirming their allocation and including contact details of the group facilitator(s). The participant will then be contacted by one of the facilitators by telephone to discuss their future involvement in the trial.

### Sample size

The primary outcome for the study is the mean SF-36 Mental Health (MH) score six months post randomisation [25]. The SF-36 MH dimension is scored on a 0 (poor) to 100 (good health) score. A previous general population survey of 3,085 Sheffield community residents aged 75 or more has demonstrated that the SF-36 can successfully be used as an outcome measure in older adults living in the community and the indications were that it was appropriate and sensitive [34]. From this general population survey, the mean SF-36 MH score was 68.3 with a standard deviation of 19.9 [34]. Differences between groups of between 5 and 10 points on the SF-36 MH score can be regarded as “clinically and socially relevant*”*[35]. The Lifestyle Matters feasibility study suggested that improvements of 7 to 14 points on the MH dimension are achievable depending on baseline functioning [36]. Assuming a standard deviation of 20 points for the SF-36 MH score at six months post randomisation, and a mean difference in MH scores between the two groups of eight or more points is clinically and practically important, then to have an 80% power of detecting this difference or more as statistically significant at the 5% (two-sided) level will require 99 participants per study arm (200 in total). However, the Lifestyle Matters intervention is a group- or facilitator led intervention. Therefore, the success of the intervention may depend on the facilitator delivering it so that the outcomes of the participants in the same group with the same facilitator may be clustered. If an average cluster size of 10 subjects per Lifestyle Matters facilitator group is assumed and an intra cluster correlation of 0.01, then the sample size must be inflated by a design effect of 1.09 to allow for this clustering giving a revised sample size estimate of 107 participants per group. Couples will be included in the trial and will count as one participant. If 20% of participants leave the study prematurely and are lost to follow-up, then it will be necessary to recruit and randomise 134 per arm (n = 268 individuals or couples (since a couple will count as one participant) in total).

### Statistical analysis

As the trial is a pragmatic parallel group randomised with a usual (control) treatment arm, data will be reported and presented according to a revised CONSORT statement [37]. Statistical analysis will be performed on an intention-to-treat-basis. All exploratory tests will be two-tailed with alpha = 0.05. Baseline demographic (for example, age, gender, number and proportion of sample who are couples) and health related quality of life data (SF-36) will be assessed for comparability between groups. The outcome data to be collected are hierarchical or multi-level in nature with individual participants nested or clustered within couples; who are nested or clustered within the Lifestyle Matters facilitation group who are then nested within a treatment group. The statistical analysis, of the outcome data, will take into account the hierarchical or clustered nature of the data by using a multi-level mixed effects linear regression model. Mixed effects models are characterised by containing both fixed and random effects. We shall assume a fixed effect for the randomised treatment group but random effects for the couple and Lifestyle Matters facilitation group. Individual participants who are not part of a couple will be treated as clusters of size one; similarly, participants randomised to the control usual care group will be treated as clusters of size one (or two if they are a couple).

To avoid bias, the independent Trial Steering Committee (TSC), the study statisticians, health economists and the research assistants collecting data at 6 and 24 months will be blinded to treatment allocation whilst the trial is ongoing. To remain blinded, assessors will not be made aware of participants’ allocation in the study and participants will be asked not to inform the assessor whether they took part in the groups when visited at 6 and 24 month follow-up. The Trial Manager, Chief Investigator, Principal Investigators, Fidelity assessment Lead, Trial Support Officer and participants will not be blinded.

#### Analysis of primary outcome

The primary analysis will compare mean SF-36 Mental Health dimension (MH) scores at six months post randomisation between the intervention group and control arms using a random-effects or multi-level mixed effects linear regression model to allow for the clustering of the outcomes within couples and Lifestyle Matters facilitation groups with baseline MH score as a covariate [38,39]. A 95% confidence interval (CI) for the mean difference SF-36 mental health dimension scores between the intervention and control arms will also be calculated. For the primary outcome, the SF-36 MH score at six months follow-up, missing data will be imputed using multiple imputations with age, gender and baseline MH scores as predictors.

#### Analysis of secondary outcomes

Secondary outcomes, such as the other dimensions of the SF-36, EQ-5D-3L, BRS, GSE, de Jong Gierveld Loneliness Scale at six months’ follow-up, will be compared between the intervention and control arm using a multi-level mixed effects linear regression model with baseline score as a covariate. A 95% CI for the mean difference in this parameter between the treatment groups will also be calculated. Participants will be followed up at 24 months post randomisation. Mean SF-36 (MH), other SF-36 dimensions, BRS, GSE, PHQ-9, EQ-5D-3L, de Jong Gierveld Loneliness Scale dimension scores at 24 months’ follow-up will be compared again using multi-level mixed effects linear regression model with baseline score as a covariate. A 95% CI for the mean difference in this parameter between the treatment groups will also be calculated.

### Cost effectiveness analysis

A trial based economic evaluation will be undertaken of an intention-to-treat comparison of the costs and outcomes of the two trial arms. A cost effectiveness analysis will be undertaken of the incremental cost per QALYs of the Lifestyle Matters intervention compared with treatment as usual [40]. QALYs will be calculated using the SF-6D preference-based index derived from the SF-36 administered at baseline, 6 and 24 months [41]. The QALY gain from the intervention will be estimated using a standard area under the curve calculation. A sensitivity analysis will be undertaken using utility values from the EQ-5D, also collected in the trial [27]. The total cost consequences of the intervention will be estimated at the individual participant level and will include the costs of providing the four month Lifestyle Matters intervention and the subsequent consequences for the use of routine health and social care services. A detailed costing of the weekly facilitated sessions will be undertaken, including recruitment (though postal invitation), administration, hire of local community venues, facilitator salaries, refreshments participant travel if required and any materials used. Care will be undertaken to exclude all research costs. Resources will be costed using local price data to estimate a total cost per session. The number of participants attending each session will be recorded and an average level of capacity used to estimate an average cost per attendance. Finally, this estimate will be applied to the actual number of sessions each participant attended.

A potentially important benefit of the intervention is that it may result in important cost savings to the NHS. The use of services by trial participants will be collected in detail using a Health and Social Care Service Use Questionnaire that will be administered by telephone or face to face. Interviewer administration is essential in order to obtain accurate and useable data on the use of all NHS and Personal and Social Services. Service use will be costed using most recent National Reference Cost Data and Unit Costs of Health and Social Care [42,43]. Missing data will be dealt with using multiple imputation for SF-6D and resource use data [44]. The central analysis of mean incremental costs per QALY will be subjected to a full sensitivity analysis of key parameters including the measure used to estimate QALYs and number participants at the weekly sessions. A full probabilistic sensitivity analysis will be performed to examine the probability of cost effectiveness of the intervention for the NHS for different levels of costs and QALY gains [45]. There will also be a supplementary cost consequences analysis that will include the other outcome measures [40].

### Maintaining participant involvement

Due to the long study period (24 months), two strategies will be employed to keep participants engaged with the trial. Firstly, a newsletter will be sent by post at approximately 7, 14 and 21 months providing information and an update on study progress. Secondly, a prize draw will take place at the end of the 6 and 24 month follow-ups for each of the three cycles at each site. Participants will only be entered into the prize draw on return of their completed questionnaires. Participants will be informed of the prize draw when they are notified of their group allocation post randomisation.

### Fidelity assessment

We will conduct a fidelity assessment to explore the appropriateness of the facilitator training, supervision and subsequent intervention adherence. Fidelity checks will assess how well the Lifestyle Matters programme is delivered according to the intervention protocol and the published manual. Checks will adhere to an intervention fidelity framework based on that identified by the Behaviour Change Consortium and NICE guidance on behaviour change [46,47]. Table 1 provides an overview of the fidelity assessment and quality assurance parameters described by Bellg *et al.* (2004), including intervention design, training, delivery, receipt and enactment [46].

**Table 1:** **Lifestyle Matters RCT fidelity assessment strategy (adapted from Bellg *et al.***[46]**)**

	**Goal**	**Description**	**Fidelity**
Trial Design	Comparable treatment	All participants have received the same programme tailored to the needs of the group/setting.	• 16 weekly meetings will be offered to all participants with delivery of a minimum of 8.
			• Four, one-to-one meetings will be offered to all participants. Uptake and attendance recorded by the facilitator.
	Risk to implementation	Plan for potential issues that could affect the delivery of the Lifestyle Matters programme.	• A range of recruitment strategies will be implemented including GP mail-outs for each geographical area, referrals from health and social care, referrals from third sector and posters/leaflets.
			• A pre-arranged set of days and times for weekly meetings will be offered from which participants can choose.
			• Undertake three recruitment cycles in three geographically separate areas, one per cycle, to prevent saturation.
Monitoring provider training	Standardised training and facilitator skill acquisition	All facilitators receive the same training programme tailored to the group/setting.	• Observation of the training session by two researchers using a content checklist (evidence of skill transference as demonstrated through, for example, role playing activities and reflective exercises).
		All facilitators understand and engage with the intervention programme training in a similar way.	• Training delivered by the same trainer.
			• Manual and CD-Rom provided to all trainees.
			• Completion of training exercises by facilitators.
Monitoring intervention delivery	Standardised delivery	All facilitators use the same techniques and content from the programme.	• Observation using a content checklist by two researchers.
			• 75% of opportunities for completing goal setting are recorded (both for individual and group).
			• Range of materials from the Lifestyle Matters programme received by all participants.
			• Facilitators maintain reflective diaries.
			• Weekly facilitator record from group meetings.
			• Participant and facilitator semi-structured interviews.
			• All participants receive certificate of attendance/achievement.
			• Facilitators meet the NHS Band 4 equivalent job description.
	Minimise drift in skills/delivery	Adherence to training content and delivery over the three cycles of the intervention.	• Observation using a content checklist by two researchers
			• OT supervisor protocol.
			• Each facilitator will attend between 8 and 16 sessions in total of which half should be delivered face-to face.
Monitoring receipt of intervention	Participant attendance and engagement	Record the numbers of participants attending the programme each week	• Registers completed by facilitators for weekly meetings and one-to-one sessions where arranged.
		All participants take part in the group meetings and activities	• 75% of opportunities for completing goal setting are recorded (both for individual and group).
		Impact of intervention on participant in terms of well-being	• Participant and facilitator semi-structured interviews.
			• Patient Reported Outcome Measures (PROMS).

The efficacy of facilitator training and supervision will be evaluated using a number of methods. All facilitators and supervisors will receive the same two day training delivered by the same trainer. The training will be observed by two participant researchers and an observation checklist will be used to evaluate delivery and receipt of the training. A purposive selection of intervention group meetings will be video recorded at each site by the facilitators. Two researchers will then assess intervention delivery using an observation checklist based on the contents of the manualised programme and two day training. Findings from the checklists will be used to identify any areas of concern regarding failure to deliver the intervention as per the intervention protocol and manualised programme. This study recognises the complexities of balancing conducting high quality research with that of delivering an intervention in a real world setting. It is appreciated, therefore, that to maintain intervention fidelity, a level of feedback is required to help develop facilitator understanding and skills during delivery of the programme based on the findings of the fidelity assessment. One of the purposes of high level research is to highlight such inadequacies, for example, in training provision that may affect intervention fidelity, but also to ensure that study fidelity is not lost. Facilitators will be asked to complete reflective diaries and a supervisor protocol will be provided as a guidance document for those involved in supervising facilitators.

A number of tools will be used to monitor participant engagement and adherence to the Lifestyle Matters programme, including attendance registers for weekly meetings and individual one-to-one sessions. Receipt of the intervention will be monitored using participant semi-structured interviews to explore perceptions and attitudes towards the programme. We will also interview at least one intervention facilitator at both sites during the first and third cycle of the intervention to elicit their experience of the training and subsequent programme delivery.

### Process evaluation

A qualitative sub-study will evaluate the impact of the Lifestyle Matters programme upon older people’s health and wellbeing and to identify factors which may mediate or moderate the effectiveness of the intervention. This will include identifying the mechanisms perceived to promote self-efficacy and resilience, evaluating the implementation of the intervention and eliciting participants’ experiences of the intervention. Semi-structured interviews will be undertaken with both participants and facilitators to explore their experience of the Lifestyle Matters intervention. Interview themes will include:

• How older people experience the programme and its delivery;

• What issues promote the effectiveness of intervention facilitation;

• The skills and competencies required to facilitate the programme;

• The barriers and facilitators to its uptake and continued use;

• The effect of the Lifestyle Matters programme on the social behaviours of older people.

All interviews lasting approximately 60 minutes will be conducted in a convenient location for the participant and audio recorded with consent. Transcripts of interviews will undergo respondent validation. This will be achieved by asking participants and facilitators to read through the transcript of their interview and comment on its accuracy. For the purposes of reporting, confidentiality will be assured by removing all identifiable or recognisable information.

Participant interviews will be conducted with a purposive sample of around 10% of participants allocated to the intervention across both sites and from all three cycles to elicit the range and nature of issues that influence their experiences of the interventions and perceived advantages and disadvantages. A sample frame will be used to identify the purposive sample which will be based on a range of characteristics, including sex, age, nationality, ethnicity, resident status, marital status, education, occupation and current levels of social and community based activity. Interviews will be conducted where possible within two weeks of attending their last group meeting.

Facilitator interviews will be conducted with at least one facilitator at each site. Because the facilitators will ideally remain the same throughout the whole study, the interviews will be conducted at the end of cycle one and cycle three. These will identify any changes in the facilitator’s experience of delivering the intervention between cycle one when they first receive and implement their training through to the third cycle when a more practiced and proficient delivery would be expected. Should there be a need for a replacement facilitator to take over a group, they will also be included in the interviews.

The following patient reported outcome measures, the Brief Resilience Scale, de Jong Gierveld Loneliness scale and the General Perceived Self Efficacy (GSE) Scale, will be used to evaluate the impact of the Lifestyle Matters programme upon resilience, self-efficacy and loneliness.

#### Analysis

Analysis of the semi-structured interviews will commence at the end of each data collection period (intervention cycle). The same methods of analysis will be applied to both the participant and facilitator interviews. Transcripts of interviews will be entered into NVivo and Framework Analysis used to examine each respondent’s data within an overall framework that is related to the intervention process. The thematic framework will be identified by two researchers and an index developed which will then be used to recode the transcripts and the data will then be charted and mapped for interpretations to develop explanations to understand the processes underlying the programme. Results will also be used to explore potential explanations for the quantitative findings and identify whether there are other emerging factors influencing uptake and impact of the intervention.

### Trial monitoring

Trial set-up and monitoring have been agreed upon with the study sponsor, the University of Sheffield. A Data Management and Monitoring Plan (DMMP) will be implemented at both sites (Sheffield and Bangor), including periodic assessment during recruitment, 6 and 24 month follow-up and study closure. Monitoring visits will include source data verification checks, data completeness checks and individual staff interviews to discuss study procedures. Additional assessments will be performed if a need is identified.

### Data management

The CTRU will undertake data management and ensure the trial is conducted according to Good Clinical Practice (GCP) Guidelines and local standard operating procedures. Data will be collected and retained in accordance with the Data Protection Act 1998. Anonymised trial data will be entered into a secure validated web based database system (Prospect) developed and hosted by the CTRU. The trial will allocate a unique screening and participant identification number to each participant. Any information provided by a participant will be handled in confidence, except where there is an issue of safety, in which the participants GP will be notified with their consent. Research participants will be protected by the removal of any recognisable, personal, confidential or sensitive data.

A requirement of the MRC LLHWB Cross-Council Programme is that primary outcome data resulting from the trial are shared with the wider research community through the UK Data Archive. Consent to share these data will be sought from participants. We will also obtain permission to share participants’ personal information with the Data Linkage Service (NHS IC), including name, address and date of birth in order to obtain regular health status reports during the period of follow-up, the purpose being, to prevent unnecessary contact with participants who may have died during this time.

### Trial oversight committees

The conduct of the trial will be overseen by three committees according to the CTRU standard operating procedures. The committees will include a Trial Management group (TMG), a Trial Steering Committee (TSC) and a Data Monitoring and Ethics Committee (DMEC). The TMG will consist of key individuals directly involved in the development and delivery of the trial including the PI, CI, Study Manager and collaborators. There will also be lay representation from an older person. The TMG will design and deliver all aspects of the trial and act on recommendations of the TSC and DMEC. The TSC will be composed of an independent chair and members with expertise in delivering RCTs and trial monitoring. The TSC will advise the CI on aspects of trial implementation, provide supervision of the trial protocol and statistical analysis plan, monitor trial progress and provide advice and consider recommendations from the DMEC. The DMEC will be composed of an independent Chair, Statistician and content expert. The DMEC is responsible for monitoring participant safety, monitoring trial results in accordance with the statistical analysis plan and providing recommendations to the TSC regarding trial continuation due to issues of ethics, safety and serious adverse events.

## Discussion

Robust and high quality research, in particular pragmatic trials in a UK setting, is required to help support service providers, health and social care managers and clinicians when making decisions about implementing evidence-based psycho-social interventions. Although Lifestyle Matters is already an established manualised programme and is being implemented sporadically in the UK by health and social care services, evidence from high level research of improved health and mental wellbeing outcomes in older people will be required before improvements in implementation will become visible. The proposed research will determine the clinical and cost effectiveness of an occupational therapy based psychosocial intervention (Lifestyle Matters) for people aged 65 years and older in a UK context, including any long term effects of sustainability.

## Trial status

Recruitment commenced on the 14 August 2012.

## Abbreviations

6CIT: Six Item Cognitive Impairment Test; AfC: Agenda for Change; BCUHB: Betsi Cadwalador University Health Board; CTRU: Sheffield Clinical Trials Research Unit; GCP: Good Clinical Practice; DMMP: Data Management and Monitoring Plan; MH: Mental health; MMSE: Mini Mental State Examination; MRC: Medical Research Council; NHS: National health service; NICE: National Institute for Health and Care Excellence; NISCHR: National Institute for Social Care and Health Research; PCRN: Primary Care Research Network; PROMs: Patient Reported Outcome Measures; QALYs: Quality Adjusted Life Years; QIPP: Quality, Productivity and Prevention; TSC: Trial Steering Committee.

## Competing interests

GM and CC are the original authors of the published Lifestyle Matters manualised programme. No other authors have any competing interests.

## Authors’ contributions

KS contributed to study design, development of trial protocol, development and preparation of supporting documents, and development of the manuscript. GM contributed to the study concept, study design, development and review of trial protocol, and the review of supporting documents. JB contributed to the study design, development and review of trial protocol, and the review of supporting documents. SC contributed to the study design, development and review of trial protocol, and the development and review of supporting documents. CC contributed to the study concept, study design, review of trial protocol and the review of supporting documents. DH contributed to the study design, development and review of trial protocol, and the development and review of supporting documents. SW contributed to the study design, development and review of trial protocol, development and review of supporting documents, and the statistical analysis plan. GW contributed to the study design, review of trial protocol, and the development and review of supporting documents. RW contributed to the study design, review of trial protocol, and the review of supporting documents. ADK contributed to the study design, development and review of trial protocol, and the review of supporting documents. TC contributed to the study design, review of trial protocol, and development and review of supporting documents. KH contributed to the review of trial protocol, and the development and review of supporting documents. All authors read and approved the final manuscript.
